# Cost effectiveness of fortified bouillon in addressing Burkinabe children's vitamin A inadequacy: An economic optimization model

**DOI:** 10.1111/nyas.15290

**Published:** 2025-02-05

**Authors:** Armando R. Colina, Stephen A. Vosti, Michael Jarvis, Reina Engle‐Stone, Aleksandr Michuda, Karen Ortiz‐Becerra, Katherine P. Adams

**Affiliations:** ^1^ Department of Agricultural and Resource Economics UC Davis Davis California USA; ^2^ Institute for Global Nutrition Department of Nutrition UC Davis Davis California USA; ^3^ Einaudi Center for International Studies Cornell University Ithaca New York USA; ^4^ Knauss School of Business University of San Diego San Diego California USA; ^5^ Banco de México Mexico Mexico

**Keywords:** Burkina Faso, costs, economic optimization, nutritional adequacy, vitamin A

## Abstract

Vitamin A dietary inadequacy remains a serious public health problem among young children 6–59 months of age in Burkina Faso. Planners face several interrelated challenges: Selecting concrete policy objectives regarding vitamin A inadequacy reductions, identifying cost‐effective vitamin A intervention programs that can achieve those objectives, and being reasonably sure that proposed intervention programs are robust to uncertainty in program benefits and costs. A 10‐year, subnational economic optimization model making use of secondary dietary intake data and program cost data was developed and implemented to address these issues and included the following vitamin A program options: existing or improved edible oils fortification, a pair of hypothetical vitamin A‐fortified bouillon programs, and a set of subnational vitamin A supplementation (VAS) programs. The model consistently identified the improved edible oils and bouillon fortification programs as the core national programs upon which the more expensive subnational VAS programs could be layered, depending on policy objectives and available funding. These results were robust to uncertainty in program nutritional benefits and costs. However, even if the most impactful set of modeled programs was implemented, vitamin A inadequacy among children would remain a serious public health problem; hence, additional efforts to address it would be needed.

## Graphical abstract

Meeting young children's vitamin A needs will require multiple programs in Burkina Faso. Economic optimization modeling suggests that large‐scale food fortification programs, especially the improved edible oils program and a hypothetical fortified bouillon cube program delivering 30% of Codex Nutrient Reference Values for adults in 2.5 g/day, form the programmatic base upon which macro‐regional vitamin A supplementation programs can be cost‐effectively layered. However, even if the most impactful programs were implemented, ∼50% of young children would still suffer vitamin A inadequacy, so additional action is needed.

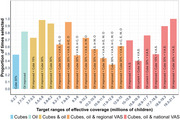

## INTRODUCTION

Vitamin A deficiency, which contributes to poor growth and cognitive development as well as morbidity among young children, continues to be a significant public health problem in low‐ and middle‐income countries (LMICs).^1^, ^2^, ^3^ In Burkina Faso, results from the 2020 National Micronutrient Survey showed that 50.2% of children 6–59 months of age suffer from vitamin A deficiency, based on serum retinol concentrations (retinol <0.70 umol/L, adjusted for inflammation).^4^ Although there are several underlying causes of micronutrient deficiencies, in most cases, dietary inadequacy is a crucial determinant of the risk of micronutrient deficiency. By this measure, household diets alone are inadequate to meet the vitamin A requirements of 94% of children 6–59 months of age in Burkina Faso. The existing edible oils vitamin A fortification program only slightly reduces that prevalence to 91%.^5^ Thompson et al. report the national effects of selected dietary inadequacies on early child mortality; an estimated 5875 child deaths in Burkina Faso (13.4% of all child deaths) each year are attributable to vitamin A inadequacy.^6^

In the long‐term, the hope is that sustainable improvements in diet quality will eventually address dietary micronutrient inadequacies and reduce the burden of deficiency; indeed, programs and policies to increase the availability and reduce the cost of nutrient‐dense foods are being undertaken or contemplated in many countries.^7^ However, such changes take time and face obstacles, so in the short term, countries have turned to large‐scale food fortification (LSFF), with particular focus on staple foods (e.g., cereal flours and edible oils) and condiments (e.g., salt), to increase the micronutrient content of diets.^8^ The impacts of LSFF programs depend on the vehicles chosen, more specifically, on the proportion of a given vehicle that is fortifiable (i.e., processed industrially), the amounts of fortificants added to premixes, the levels of consumption (portion sizes, essentially) by at‐risk populations, and hence the amounts of micronutrients consumed by targeted beneficiaries. Bouillon, a commonly consumed condiment in West Africa, has recently been added to the list of candidate micronutrient delivery vehicles alongside existing LSFF programs.^9^, ^10^ Bouillon is especially noteworthy because of its extensive reach, geographically and across socioeconomic groups, in some countries. In Burkina Faso, bouillon was reportedly consumed by 82% of households (ranging from 70% in the Cascades Region to 95% in the Sahel), and by over 80% of poor households compared to roughly 70% of relatively well‐off households.^5^

Finally, regarding programs available to address vitamin A inadequacy among young children, vitamin A supplementation (VAS) programs can be an effective and geographically targetable tool. However, as demonstrated below, these programs can be costly and, hence, their sustainability is concerning.^11^

Therefore, resource‐constrained planners face several interrelated challenges in choosing among the policy options available to them. First, what should be the targeted level of vitamin A inadequacy reduction, that is, how much progress can or should we aim to make in addressing this problem? Second, as financial resources will always be limited, what set of vitamin A programs can achieve alternative targeted levels of vitamin A inadequacy reduction at the lowest cost? Third, given the uncertainty in program impacts on vitamin A inadequacy and program costs, can we be reasonably sure that the proposed lowest cost set of vitamin A programs is the best option available?

This paper addressed these issues in the context of Burkina Faso by bringing together modeled evidence on levels of vitamin A inadequacy among children 6–59 months of age and on the costs and impacts on vitamin A inadequacy of alternative existing and hypothetical LSFF and VAS programs. We paid particular attention to bouillon fortification with different amounts of vitamin A, and to the costs that would be faced by governments and industry (the set of implementing agents truly responsible for food/condiment fortification). We developed and used a multi‐period economic optimization model that includes each vitamin A program and all possible combinations of programs over a 10‐year planning time horizon. Each program is characterized by annual start‐up (as relevant) and operational costs faced by governments and industries, and an annual stream of children who achieve vitamin A adequacy over the 10‐year horizon due to the program. The optimization procedure searches over individual vitamin A programs, and all possible combinations of vitamin A programs, to find the least costly program packages (comprising one or more programs) given alternative policy targets for vitamin A adequacy among children. Resource‐constrained planners will find the results useful for selecting among and designing programs that meet their objectives for reducing vitamin A inadequacy among children, for identifying program packages that can affordably and efficiently meet them, and for developing a better understanding of how LSFF programs, and especially bouillon cube fortification programs, fit into a national strategy for addressing vitamin A inadequacy.

## MATERIALS AND METHODS

### Overview

Our main objective was to identify the least costly vitamin A program packages for alternative target levels of vitamin A adequacy among children in Burkina Faso. The economic optimization model set out below was designed to choose from among all the available programs and combinations of them to find the least costly program packages for achieving target levels of effective coverage, that is, the number of children who shift from vitamin A inadequacy to vitamin A adequacy due to the program or program package selected. The model does so based on the modeled annual streams of nutritional benefits (measured as children effectively covered) and costs associated with each program or package of programs, with the important caveat that program costs can be summed across programs, but the same is *not* true for nutritional benefits.^a^

### Spatial scope and aggregation

**FIGURE 1:**
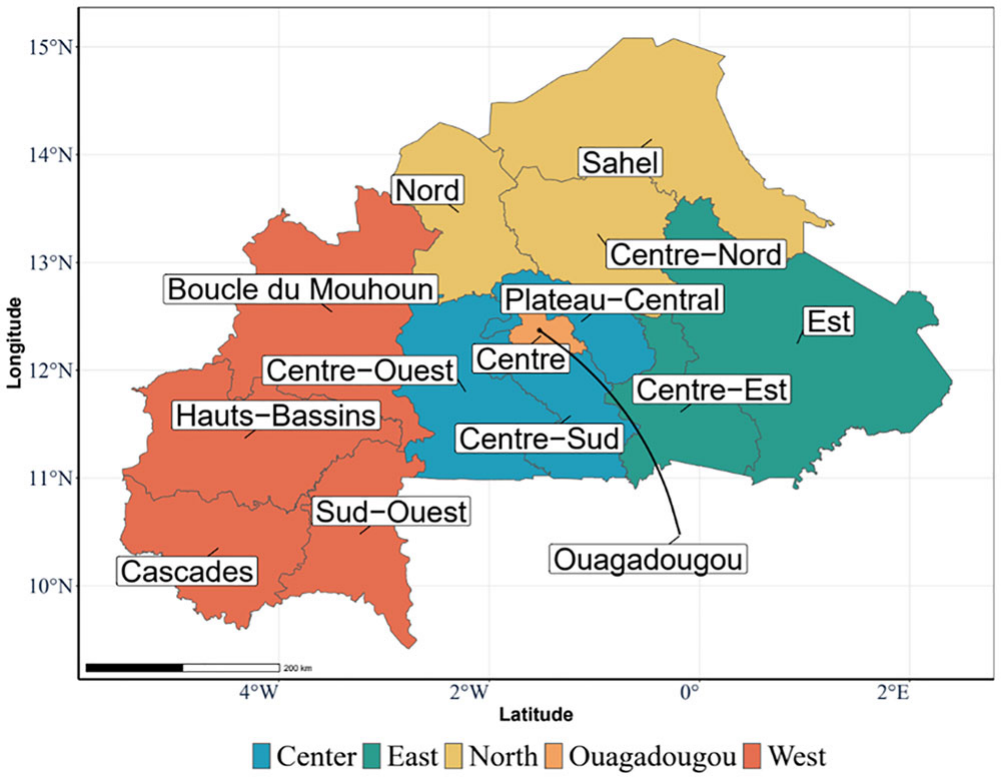
Regions and constructed macro‐regions of Burkina Faso.

We aggregated the country's regions into five agroecological/economic macro‐regions, including the major metropolitan area of Ouagadougou (Figure 1).^b^ These macro‐regions were delineated by in‐country collaborators familiar with vitamin A deficiencies and programs developed and implemented to address them.

### Target population

We focused exclusively on children 6–59 months of age. Table 1 identifies the five macro‐regions used in this analysis, the formal administrative regions that they comprise, the number of children 6–59 months of age in the model's baseline year (2023) in each macro‐region, and the percentage of children with dietary vitamin A inadequacy^c^ in each macro‐region based on dietary intake alone.^d^ Nationally and subnationally, dietary vitamin A inadequacy among young children is clearly highly prevalent.

**TABLE 1:** Child populations and vitamin A inadequacy over time, by macro‐regions in Burkina Faso.

Macro‐regions	Administrative regions	Child population		% Vitamin A dietary inadequacy^a^
		2023	2033^b^	2023–2033^a^
Ouagadougou	Ouagadougou	464,940	546,000	88%
Center	Centre‐Ouest, Centre‐Sud, Plateau‐Central	552,273	648,559	96%
North	Centre‐Nord, Nord, Sahel	753,577	884,960	99%
East	Est, Centre‐Est	542,860	637,506	88%
West	Boucle‐de‐Mouhoun, Hauts‐Bassins, Cascades, Sud‐Ouest	933,013	1,095,680	95%
**Total**		**3,246,663**	**3,812,705**	**94%**

^a^ Estimates from Adams (2024) based on the nutrient density of the household diet compared to the critical vitamin A density of children, estimated using household food consumption data from the 2018–2019 Enquête Harmonisée sur les Conditions de Vie des Ménages (EHCVM).^5^, ^14^ We assume that diets do not change over the 10‐year modeling time horizon, so the % vitamin A Inadequacy remains the same but is applied to a larger child population in 2033. The contributions of vitamin A supplementation program are not included in this assessment.

^b^Regional population projections based on national UN World population prospects, 2019 update, and weighted by 2020 projected regional population shares accordingly.^13^ Macro‐region population estimates based on summing of regional projections.

### Policy choices

Policy modeling requires clearly defined policy instruments. Table 2 identifies the vitamin A programs to be considered, individually and jointly; see Table S1 for more detail on the mandated and assumed fortification levels and industry compliance.^15^ All vitamin A LSFF programs must be implemented at the national level (or not at all), whereas VAS programs can be spatially targeted at the macro‐region. Improvements in existing LSFF programs are possible, for example, the most recent evidence suggested that only 39% of fortifiable edible oils are fortified, and among fortified edible oils, the average fortification level was 85% of the national standard of 17.5 mg/kg.^5^, ^16^ However, investments could hypothetically be made to improve this program performance to an assumed 75% of the national standard (i.e., 75% of fortifiable edible oils fortified to 100% of the national standard).^e^ Although there may be some voluntary fortification of bouillon cubes with vitamin A in Burkina Faso, a national bouillon cube fortification program is hypothetical.^f^ That said, bouillon cube consumption is widespread geographically and across socioeconomic groups in Burkina Faso, and the focus of national fortification policy discussions in Nigeria.^17^ Therefore, we modeled two bouillon cube fortification program options operating at the same assumed level of performance (75% of bouillon cubes fortified to the hypothetical standard): (i) one fortified at a level to provide 15% of Codex Nutrient Reference Value (NRV) for vitamin A to an adult consuming 2.5 g/day of bouillon cube (corresponding to 48 mg vitamin A per kg bouillon) and (ii) one fortified to provide 30% of that same NRV for vitamin A in 2.5 g (corresponding to 96 mg vitamin A per kg bouillon).

**TABLE 2:** Vitamin A programs available to planners.

Program description	Program label	Program scope	
		*National*	*Subnational*
Current edible oils program (39% compliance^a^)	Oil current	X	
Improved edible oils program (75% compliance)	Oil improved	X	
Bouillon cube at 15% of Codex NRV in 2.5g	Cube 15%	X	
Bouillon cube at 30% of Codex NRV in 2.5g	Cube 30%	X	
VAS in Ouagadougou	VAS in O		X
VAS in Center macro‐region	VAS in C		X
VAS in North macro‐region	VAS in N		X
VAS in East macro‐region	VAS in E		X
VAS in West macro‐region	VAS in W		X

Abbreviations: NRV, nutrient reference value; VAS, vitamin A supplementation.

^a^“Compliance” refers to the proportion of the fortifiable foods/condiments produced by industry that is fortified. Compliance estimates are taken from Adams et al. (2024).^5^

### Nutritional benefits

The measure of nutritional impact used in this analysis is effective coverage, which, by definition, is the number of children who achieve dietary vitamin A adequacy due to one or more of the vitamin A programs being considered. To compute the benefits of each program, we first used household‐level food consumption data from the 2018–2019 Enquête Harmonisée sur les Conditions de Vie des Ménages (EHCVM)^14^ to estimate the vitamin A adequacy of diets without vitamin A programs and then to model changes in the vitamin A adequacy of diets with alternative LSFF programs and/or high‐dose VAS. Methods are described in detail in Adams et al. ^5^

In short, to estimate the micronutrient content of foods, we matched food items in the EHCVM food consumption and acquisition module with entries from the West African Food Composition Table, supplemented with entries from the Nutrition Data System for Research and the Malawian food composition table.^18^, ^19^, ^20^, ^21^ Then, based on respondent recall of the quantity of each of the 138 food items consumed by household members in the 7 days prior to the survey, we estimated the total daily apparent household intake of vitamin A and energy. We converted these data points into estimates of the vitamin A density of the household diet by dividing the total daily apparent vitamin A intake by total daily apparent energy intake, expressed in per 1000 kcal calories (kcal) terms.^22^ To estimate the prevalence of vitamin A inadequacy without programs, we then compared the vitamin A density of the household diet to critical vitamin A densities for children 6–59 months of age, where the critical vitamin A density is the child's age‐ and sex‐specific estimated average requirement divided by his/her age‐ and sex‐specific energy requirement, expressed in per 1000 kcal terms. Note that we assessed adequacy using the energy‐adjusted nutrient density of the household diet in an effort to address some of the error inherent in household‐level food consumption estimates that result from recall error and inadequate accounting for foods consumed away from home.^23^

To model the addition of vitamin A delivered by edible oil fortification, bouillon cube fortification, or both of these LSFF programs, we multiplied the total household daily apparent consumption of the relevant food vehicle by the estimated average fortification level and then recalculated the nutrient density of the household diet and prevalence of inadequacy.

Burkina Faso also has a national high‐dose VAS program for children 6–59 months of age.^g^ Except for children nine months of age, VAS was assumed to be received via a national campaign. For the Plateau Central, Centre‐Ouest, Sud‐Ouest, Centre Sud, and Hauts‐Bassins regions, VAS coverage in urban and rural areas via national campaign was based on the second round of the 2020 Post‐Campaign Coverage Assessment Survey of National Vitamin A Supplementation Days. For all other regions, campaign coverage in urban and rural areas was based on the 2018 Report of the Evaluation Survey of the National Post‐Campaign Coverage of VAS, Deworming and Malnutrition Screening. For children 9 months of age, VAS was assumed to be received at a routine clinic visit, and coverage was based on measles vaccination coverage rates according to the 2021 Demographic and Health Survey.^24^ See Table S2 for modeled VAS coverage data and assumptions. To model the impact of VAS, we randomly assigned children within each region in the EHCVM data to these region‐specific coverage levels. That is, in proportion to the region‐specific coverage level (Table S2), we randomly assigned children to receive VAS or not. Following Engle‐Stone et al., receipt of VAS was converted to a daily equivalent intake of 167 µg retinol activity equivalents (RAEs)/day.^25^ This daily equivalent value (along with other sources of dietary vitamin A, including that provided by LSFF programs, where relevant) was incorporated into the vitamin A density of the diet and compared to the vitamin A requirements of children 6–59 months of age.

We estimated the proportion of children effectively covered as the change in the prevalence of vitamin A inadequacy among children 6–59 months of age with individual or combined vitamin A intervention programs compared to baseline diets without programs. We translated these into the number of children effectively covered by multiplying the prevalence of effective coverage by the population of children 6–59 months of age in each year of the modeling time horizon.^h^

### Vitamin A program costs

We used an ingredient‐ and activity‐based approach to estimate the economic costs of planning, designing, launching, and operating vitamin A programs in Burkina Faso. Cost estimates were generated for the three key stakeholder groups associated with LSFF programs, namely, government, industry, and consumers (to whom premix and other fortification program costs are passed via increases in the prices of fortified foods). However, because all LSFF programs are industry‐based and are designed and managed by government, our main perspective was on governmental and industry costs, net of premix costs.^i^ This approach has a long history in private‐ and public‐sector analyses and in the economic evaluation of nutrition intervention programs and VAS programs.^26^, ^27^, ^28^, ^29^, ^30^ This approach focuses exclusively on the marginal costs of fortification for governments and industry, that is, we did not address the costs of other ingredients (e.g., edible oils, salt, and flavoring) and activities (e.g., overall factory management) required to produce fortifiable foods/condiments. However, in the case of VAS programs, all planning, operational, and capsule costs are included. It is important to note that the time costs to caregivers seeking VAS for their children are not directly included in this analysis but are reflected in the VAS coverage data used to estimate both the nutritional benefits and the costs of programs and program packages.^j^

We estimated the costs of planning and launching new programs and the cost of redesigning and re‐launching improved existing programs. In the context of new programs, changing a factory production line or launching a new government program requires investments. For example, food manufacturers’ costs include buying new production and laboratory equipment, training personnel in their use and maintenance, and preparing for additional internal quality assurance and quality control activities. For government agencies, the start‐up costs include social marketing for the program, planning for the program's deployment, and training government regulatory and other staff. The delivery‐vehicle‐specific cost models were comprised of these investments and activities. In the context of improvements to existing programs, in this case improvements to the existing edible oils program, training and outreach investments and increased outlays for internal factory monitoring and quality assurance are required by industry, and enhancements are required in factory and import monitoring on the part of the government. Estimates of the cost of these improvements were based on interviews with industry members and in‐country experts on food fortification programs and possible improvements to them. Additionally, previous estimates of fortification program improvements from another West African country (Cameroon) were used as a general guideline when determining the amounts and types of costs required to bring about and sustain program improvements.^29^

We also estimated recurring costs for all actors involved in LSFF programs throughout the 10‐year modeling time horizon. For example, we estimated the additional costs of labor, as well as the costs of performing internal and external quality controls, that manufacturers would need to incur to comply with the fortification program standards. For government agencies, we estimated the costs of monitoring factories and imports for quality assurance and of social marketing activities.

Burkina Faso currently imports all of its bouillon products. Still, fortification standards apply to domestic *and* international producers, so the government must monitor and regulate the flows of bouillon products entering the country. Therefore, these and other costs associated with bouillon cube fortification were explicitly included in the LSFF program cost models, regardless of where that fortification occurs.^k^

VAS program costs comprised the costs of two VAS delivery platforms—campaign‐based distribution and clinic‐based distribution, which are currently operating in Burkina Faso. Activity‐based cost models were developed for each delivery platform based on in‐country records/budgets of previous VAS programs, estimates provided by individuals associated with supplementation programs in Burkina Faso, and other models previously developed for supplementation platforms.^30^ The platform‐specific shares of VAS coverage were used to estimate the total cost of VAS programs for each macro‐region (see Table S2 for underlying data and methodological details).^l^

LSFF program and VAS program cost models were designed to reflect a 10‐year planning time horizon, with start‐up costs occurring during the first 2 years for hypothetical bouillon cube fortification programs and during the first year for the improved edible oils program; operational periods continue for 8 and 9 years, respectively. VAS programs face only annual, macro‐region‐specific operational costs primarily driven by personnel, capsule, and distribution costs.^m^

Finally, regarding vitamin A program costs, the size and age structure of the national population, and hence the consumer bases for fortified food vehicles and VAS, were the only cost model parameters that varied over time; all other technical parameters and unit values (e.g., wage rates) were held constant throughout the 10‐year model time horizon.

### Economic optimization model

We used linear programming techniques to solve an optimization problem from the point of view of a program planner.^n^^,^ o This planner was assumed to focus on government and industry costs, to have limited financial resources, and to have many individual vitamin A programs and combinations of vitamin A programs to choose from. We assumed that the planner had a planning time horizon of 10 years, a policy target regarding the minimum number of children to effectively cover over that time horizon and then sought to identify the program package (comprising one or more vitamin A programs) that achieved the target level at the lowest cost. We solved the optimization problem for various alternative target levels of effective coverage and found the least costly program or program package associated with each target level of effective coverage. The planner can always choose the status quo vitamin A fortification program (the current, relatively low‐performing edible oils vitamin A fortification program).^p^ However, given that 91% of children remain inadequate in vitamin A with that program in place, we use the model to explore lower target levels of inadequacy.^q^

As indicated above, the planner has available several vitamin A programs and combinations of them. Recall that VAS programs can target one or more specific macro‐regions of the country. In contrast, all LSFF programs must be implemented at the national level because of the scale of industrial fortification (internationally or domestically produced) and the challenges associated with maintaining a level commercial playing field for vitamin A delivery vehicles due to porous intra‐national borders.^33^

Each vitamin A program has a schedule of benefits and costs throughout the 10‐year planning horizon. As previously noted, all new programs have start‐up costs: For bouillon, government and industry will incur these up‐front costs, and the nutritional benefits will accrue (completely and immediately) in year three and will rise in proportion to the growth of the child population until the end of the planning time horizon. The costs associated with improving the edible oils program are paid in year 1, and the marginal nutritional benefits of these investments begin to accrue in Year 2. To capture the cost of waiting for the nutritional benefits to accrue and the sense of urgency associated with solving vitamin A inadequacy problems sooner rather than later, we use a discount rate of 3% per year for nutritional benefits. Similarly, to account for the time value of money and for the relatively high up‐front costs faced by government during the start‐up period,^30^ we apply the same discount rate to costs.

### Model specification

The planner faces two menus of vitamin A programs, one menu contains national programs, $N=\{N_{1},N_{2},\text{…},N_{k^{N}},\}$, and the second menu consists of macro‐regional VAS programs $S=\{S_{1},S_{2},\text{…},S_{k^{S}},\}$.^r^ There are many programs or combinations of programs that could be chosen, and the model is designed to choose the least costly combination of national and subnational programs that achieve a target level of effectively covered children over a 10‐year span. We refer to each possible combination of programs as a program package.

More specifically, programs $N_{k}$ and $S_{k}$ are binary variables that take on a value of one if they are chosen to be implemented, and zero otherwise. For example, one possible program package could be $\left\{N^{*},S^{*}\right\}=\left\{N_{1}=1,N_{2}=1,N_{3}=0,\text{…},N_{k^{N}}=0,S_{1}=1,S_{2}=0,S_{3}=0,\text{…},S_{k^{S}}=0\right\}\;$, that is, national programs #1 and #2 are chosen but not national program #3 or the other $k^{N}-3$, whereas macro‐regional program #1 is chosen but not the other $k^{s}-1$ options.

We constructed two menus of programs, the first includes four national LSFF programs, and the second menu includes six macro‐regional VAS programs (five macro‐regional VAS options, plus the option of selecting all macro‐regions for a nationwide VAS program; see Table 2 for further details). The planner can choose a stand‐alone national oil or cube program, or combine either national oil program with either national cube program. This leads to eight possible combinations. Furthermore, the planner can choose to combine any of these eight options with regional VAS programs in one, two, three, four, or five of the macro‐regions. For example, the planner could consider the Oil current program in conjunction with a cube 30% program, complemented with a VAS program in the Center and East macro‐regions. The planner could also consider adding the West and/or the North macro‐regions. This leads to 287 alternative program packages that can be selected from either or both of the national and macro‐regional program menus, each generating unique costs and nutritional benefits.

Recall that the impacts of vitamin A programs on nutritional benefits, here and throughout defined as the number of effectively covered children per year, $EF_{t}\left(\left\{N^{*},S^{*}\right\}\right)$, are not linearly additive.^s^ Therefore, a program package consisting of two programs will not necessarily yield the same level of benefits of the sum of those two programs. For example, suppose $\hat{N}=\left\{\;N_{1}=1,\;N_{2}=1,\text{…},N_{k^{N}}=0\right\}$, $N^{\prime }=\left\{\;N_{1}=1,\;N_{2}=0,\text{…},N_{k^{N}}=0\right\}$, and $N^{\prime \prime }=\left\{\;N_{1}=0,\;N_{2}=1,\text{…},N_{k^{N}}=0\right\}$. Then, $EF_{t}\left(\left\{\hat{N},S^{*}\right\}\right)\;\text{…}EF_{t}\left(\left\{N^{\prime },S^{*}\right\}\right)+EF_{t}\left(\left\{N^{\prime \prime },S^{*}\right\}\right)$.

Again, we assume that implementing a new bouillon cube fortification program (regardless of the premix choice) requires a 2‐year set‐up period during which program establishment costs have to be paid but no nutritional benefits are generated. The start‐up period for the improved edible oils program is 1 year; VAS programs face no start‐up costs because they are already in place. Once programs are established, nutritional benefits in terms of effective coverage begin to flow and are accounted for in the number of effectively covered children per year, $EF_{t}\left(\left\{N^{*},S^{*}\right\}\right)$.

Each combination of programs generates yearly costs, $\;C_{t}\left(\left\{N^{*},S^{*}\right\}\right)$. We assume that costs are linearly additive across programs.^t^ We also assume that the planner has a 10‐year planning horizon and seeks to use available resources as efficiently as possible while reaching desired alternative target levels of effective coverage, $\bar{EF}$. Therefore, the planner solves the following optimization problem for each level of target effective coverage^u^:

$$\min_{\left\{N,S\right\}}\sum_{t=1}^{10}\frac{C_{t}\left(\left\{N,S\right\}\right)}{{\left(1+r\right)}^{t-1}}$$

(1) $$s.t.\sum_{t=1}^{10}\frac{EF_{t}\left(\left\{N,S\right\}\right)}{{\left(1+r\right)}^{t}}\geq \bar{EF}$$

Recall from Table 1 that in 2023 there were approximately 3.2 million children, 93.7% (∼3.1 m) of whom suffered from dietary vitamin A inadequacy—we use the model to find cost‐minimizing program packages to systematically reduce that burden. Technically, in what follows, we use the model to identify the least costly program packages associated with 40 alternative target effective coverage levels, ${\bar{EF}}_{l}\equiv \left\{{\bar{EF}}_{1},{\bar{EF}}_{2},\text{…},{\bar{EF}}_{40}\right\}$, beginning at a very low level of targeted effective coverage representing ∼1% of the VA‐inadequate children population, and increasing target level of effective coverage by increments that correspond to ∼2.5 percentage points of the population of children with vitamin A inadequacies.^v^

### Introducing uncertainty in intervention program costs and benefits

Finally, in terms of methods, the outcomes of the nutritional benefits and costs models represent our central expectations of the nutritional benefits and costs of the different programs and program packages assessed over a 10‐year planning time horizon. However, food and condiment market conditions, dietary intake patterns, and other factors related to micronutrient intervention program costs and their nutritional benefits may change considerably over the model's 10‐year time horizon.^w^ These changes may lead to the identification of different sets of cost‐effective vitamin A programs or program packages from those identified by the non‐stochastic model identified above.

To test for the sensitivity of the economically optimal solutions to differences in costs and nutritional benefits, we performed simulations in which costs and nutritional benefits deviated from our central estimates.^x^ More specifically, we assumed that the potential deviations from the central estimates of nutritional benefits and costs are normally distributed with mean zero and standard deviation of 0.1 (i.e., N(0,0.1)), and then, we performed 480 simulations by taking independent draws from this distribution. In each round of simulations, the new benefits and costs of each program were equal to the central estimates plus the drawn deviation.^y^ This implies that in 68% of the simulated scenarios, the costs and nutritional benefits will be within ±10% from our main estimates and in an additional 27% of the scenarios the shocks imply changes between ±10% and ±20%. For example, it is possible that in one round of simulations the nutritional benefits of the VAS program in Ouagadougou increased by 10% and its costs by 3%; in that same simulation, the nutritional benefits of implementing fortified bouillon cube at 15% of Codex NRV in 2.5 g were 12% lower and its costs 19% higher.

## RESULTS

**FIGURE 2:**
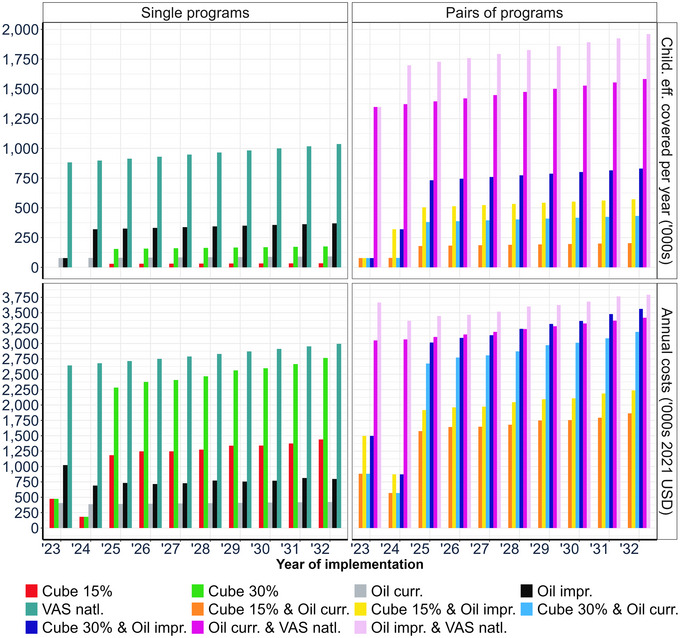
Annual flows of children effectively covered and program costs for selected vitamin A programs and program packages.

Figure 2 (top row) depicts the annual flows of nutritional benefits, in terms of children effectively covered,^z^ for individual vitamin A fortification and supplementation programs and combinations of them.^aa^ Nutritional benefits of all programs and combinations of them trend up over time as the population of young children grows. Note that there is a 1‐year delay in the flow of benefits of the improved oil (oil imp) program and a 2‐year delay in nutritional benefits flows for the bouillon fortification programs; these delays are attributable to program start‐up costs. Note also the substantial differences in the levels of annual benefit flows across the individual programs (e.g., the current vs. the improved oil fortification programs) and across the combinations of programs (e.g., the fortified bouillon at 15% of Codex NRV plus the current oil fortification programs vs. the current oil fortification program plus the national VAS). Finally, the national VAS program stands out as a potentially huge contributor to reducing vitamin A inadequacy, but the costs of this and other programs also need to be considered.

Figure 2 (bottom row) reports the annual costs of the same subset of vitamin A programs, and selected pairs of them, considered in this analysis. Costs also trend upward over time, again owing to the larger population of young children over the model simulation period. Note that the program packages that included fortified bouillon cubes faced substantial start‐up costs during the first 2 years of the model simulation period. There are very substantial differences in program costs (e.g., bouillon cubes fortified at 15% vs. 30% of Codex NRV). Once again, a national VAS program stands out, but in this case by being the most expensive vitamin A program every year.

### Summary economic optimization model results

**FIGURE 3:**
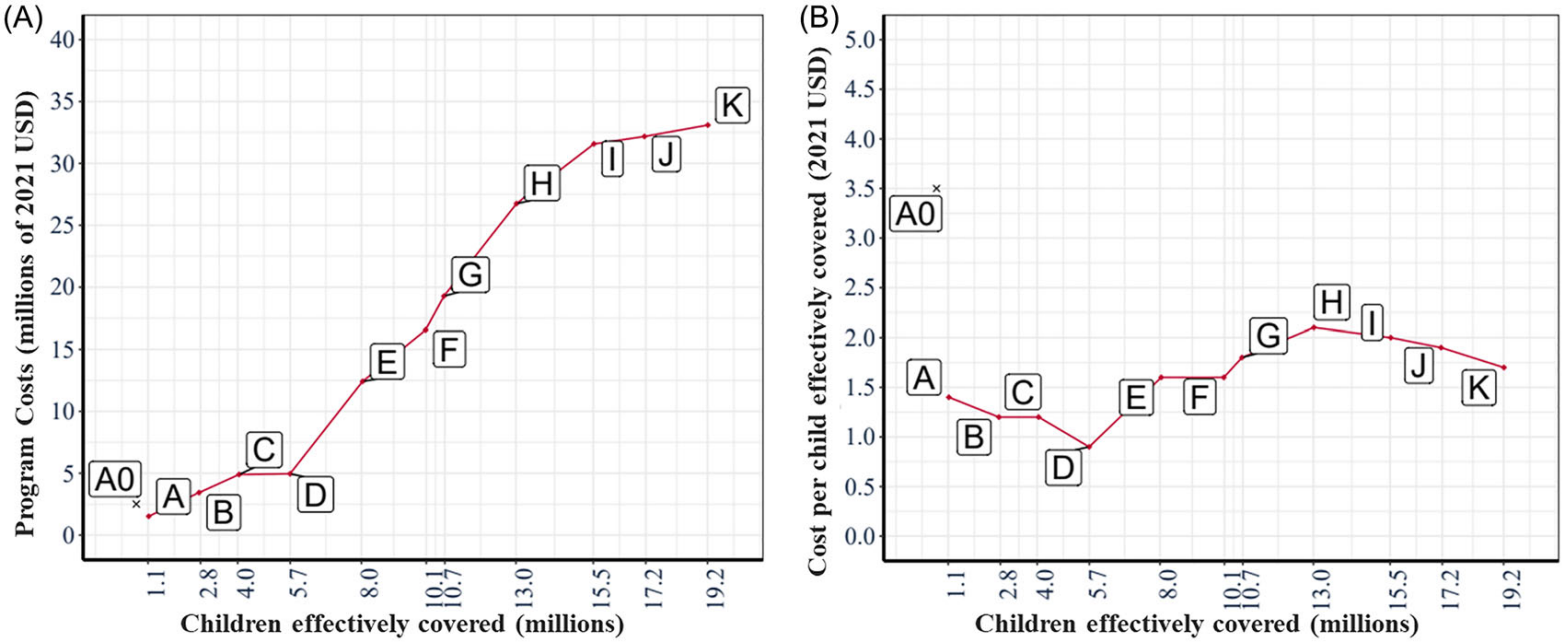
Total costs (A) and cost effectiveness (B) of economically optimal vitamin A programs and program packages. Scenario labels A through K represent alternative sets of optimal vitamin A intervention programs; see Table 3. Scenario A0 represents the (benchmark) current vitamin A–fortified edible oils program.

We present the results of our optimization modeling using our central expectations of future benefits and costs in Table 3 and Figure 3A,B. Table 3 reports the summary results of economically optimal programs and program packages for increasing target levels of effective coverage. In column 1, we report the labels (A through K) of the sequence of vitamin A programs or program packages that are economically optimal for each target level of effective coverage. The current or baseline program being implemented in Burkina Faso consists of fortified oil with 39% compliance (labeled A0). Columns 2 and 3 of Table 3 report, respectively, the labels of the specific national LSFF and macro‐regional VAS programs and program packages that met each row's increasing (reading from top to bottom) target levels of effective coverage at the lowest cost. For example, in scenario C, the improved edible oils and fortified bouillon (at 15% of Codex NRV) programs comprised the least costly program package for effectively covering 4.0 million children over a 10‐year period. Column 4 reports the number of children effectively covered^bb^ and column 5 the percentage of all vitamin A–inadequate children that each absolute number represents; for example, program package C (oil improved + cube 15%) effectively covers 4.0 million children over 10 years, which represents approximately 10.5% of the total number of vitamin A–inadequate children.

**TABLE 3:** Economically optimal vitamin A programs and program packages for different target levels of effective coverage over 10 years.

Labels^a^	Optimal VA intervention programs and program packages	VAS macro‐regions	Children effectively covered		Total cost^b^	Cost/child effectively covered
(1)	(2)	(3)	(4)	(5)	(6)	(7)
			*(Millions)*	*(Percentage over ten years)*	*(Millions of USD)*	*(USD)*
A0	Oil current		0.7	1.8%	2. 5	3.5
A	Cube 30%		1.1	2.9%	1.5	1.4
B	Oil improved		2.8	7.3%	3.4	1.2
C	Oil improved + cube 15%		4.0	10.5%	4.9	1.2
D	Oil improved + cube 30%		5.7	15.0%	5.0	0.9
E	Oil improved + cube 30% + VAS	E, O	8.0	21.0%	12.4	1.6
F	Oil improved + cube 30% + VAS	W, O	10.1	26.5%	16.6	1.6
G	Oil improved + cube 30% + VAS	C, W	10.7	28.1%	19.3	1.8
H	Oil improved + cube 30% + VAS	C, E, W, O	13.0	34.1%	26.7	2.1
I	Oil improved + VAS	C, E, W, O, N	15.5	40.7%	31.6	2.0
J	Oil current + cube 30% + VAS	C, E, W, O, N	17.2	45.1%	32.2	1.9
K	Oil improved + cube 30% + VAS	C, E, W, O, N	19.2	50.4%	33.1	1.7

Abbreviations: E, East macro‐region; N, North macro‐region; O, Ouagadougou macro‐region; VAS, vitamin A supplementation; W, West macro‐region.

^a^Column 1 labels identify programs and program packages in subsequent figures.

^b^Total costs represent the 10‐year sum of start‐up and non‐premix costs of all programs.

Column 6 of Table 3 reports the total cost (including start‐up and operational costs) associated with the baseline program and the program packages identified as economically optimal. Finally, column 7 of Table 3 reports the average cost per effectively covered child over the model's 10‐year planning time horizon for the baseline program (A0) and for each of the economically optimal programs and program packages. Note that the baseline program, A0, effectively covers 0.7 million children through a 10‐year span and is more expensive than the first optimal intervention, fortified cubes at 30% of Codex NRV. Similarly, the average cost per child effectively covered is noticeably higher in the current oil fortification program than all of the programs and program packages that are deemed economically optimal.

Several interesting patterns emerge from the results reported in Table 3. First, at low levels of targeted effective coverage (and consequently at low levels of program costs), the LSFF programs emerged as the most efficient options. Second, these programs remained part of all optimal intervention packages as target levels of effective coverage increased. Third, macro‐regional VAS programs varied in efficiency, so if funding were limited, these could be targeted at more cost‐effective macro‐regions, beginning with the East and Ouagadougou (scenario E).

Figure 3A,B depicts key results reported in Table 3. In Figure 3A, we see that the first economically optimal program packages, labels A through D, consist of the relatively inexpensive LSFF programs costing up to $5 million over 10 years and effectively covering up to approximately 5.7 million children. To effectively cover more children, the economically optimal program packages require spending substantially more, mainly on combinations of macro‐regional VAS programs. The largest number of children that could be effectively covered by the most impactful program package was approximately 19.2 million over 10 years at a cost of ∼33.1 million USD. That is to say, even if the most impactful program package included in this modeling exercise were deployed, only approximately half of the approximately 35.8 million children (in 2033) who were vitamin A–inadequate in Burkina Faso would achieve vitamin A adequacy.

Figure 3B depicts the relative cost effectiveness of the economically optimal program and program packages associated with scenarios A through K, progressing from the lowest level of impact to the highest. Initially, cost per child effectively covered declines as we move from Scenarios A to D; the economically optimal sequence of LSFF programs and program packages generally become more cost‐effective at reducing vitamin A inadequacy among children. Cost per child effectively covered rises markedly as the first VAS program is introduced (scenario E), drifts upward to the program package associated with scenario H as increasingly less efficient macro‐regional programs are added, and then gradually falls as VAS programs become national in scope, and the most efficient LSFF programs are introduced alongside them.

### Robustness to changes in future costs and benefits

A perennial issue in policy analysis is the extent to which uncertainty in program benefits and/or costs might influence the program packages that emerge from the economic optimization model. To test the robustness of the economically optimal program packages in Table 3, we introduce program‐specific uncertainty in benefits and costs.

**FIGURE 4:**
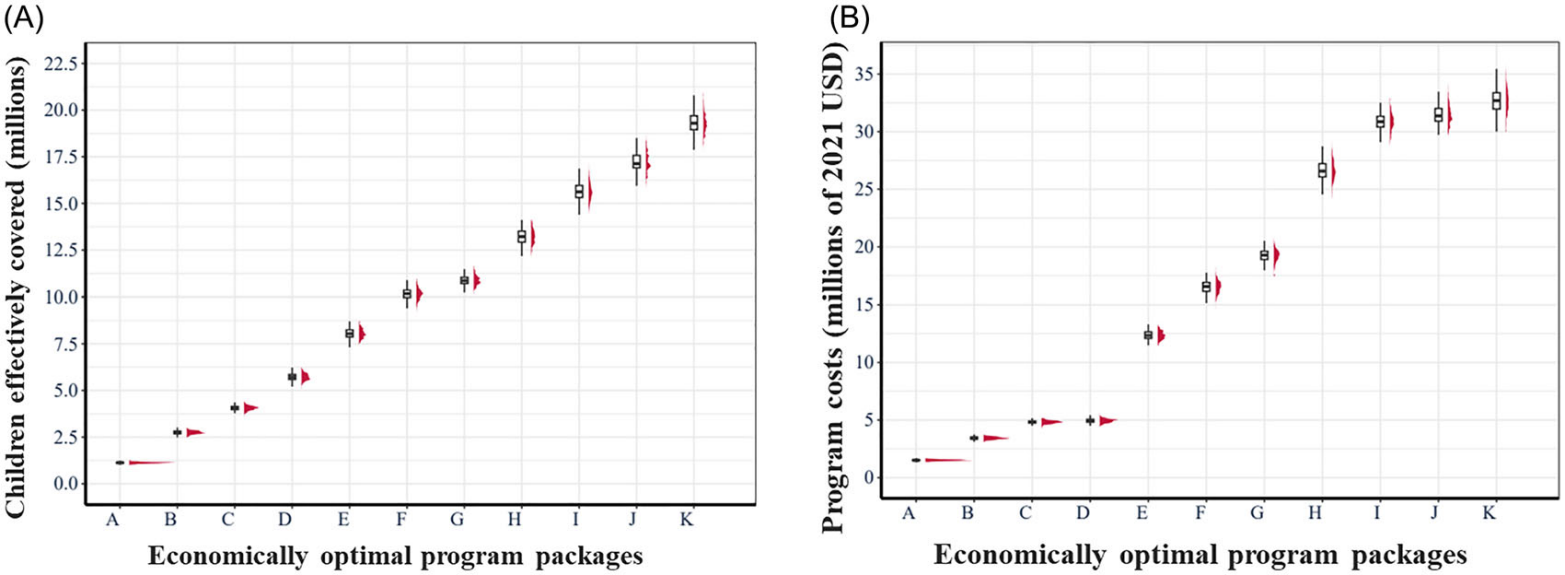
Ranges and distributions of children effectively covered and costs of vitamin A programs and program packages deemed economically optimal under central estimates. (A) Distributions of the total number of children effectively covered under stochastic benefits. (B) Distribution of the total costs under stochastic costs. The labels A through K represent different economically optimal programs and program packages, as identified in Table 3. The whisker diagrams associated with each program or program package depict the mean, and the first and third quartiles of the marginal distribution. The red masses to the right of each whisker diagram depict the marginal distributions of benefits or costs.

In Figure 4A,B, we report the ranges (whisker diagrams) and distributions (red figures to the right of each whisker diagram) of nutritional benefits and costs of the programs and program packages originally deemed economically optimal under our central estimates (as reported in Table 3). Across the 480 simulations, an interesting feature is that the variance of benefits and costs of the programs and program packages tends to increase as more programs are included. For relatively low levels of effective coverage, we observe little to no overlap in the distributions, especially for the distribution of benefits. However, for higher levels of effective coverage, we observe that the distributions of benefits and costs become wider and flatter. In many cases at high levels of targeted effective coverage, there is considerable overlap between the program packages deemed optimal under our central estimates. For example, on average, program package G yields a higher level of effective coverage than program package F, but in many simulations, their benefits would be similar at ∼11 million children effectively covered over 10 years. Similarly, program packages that were deemed optimal that include macro‐region–specific VAS programs show large and overlapping dispersions in both benefits and costs. This raises the question of how robust the economically optimal solutions are as we move along the continuum of targeted effective coverage.

**FIGURE 5:**
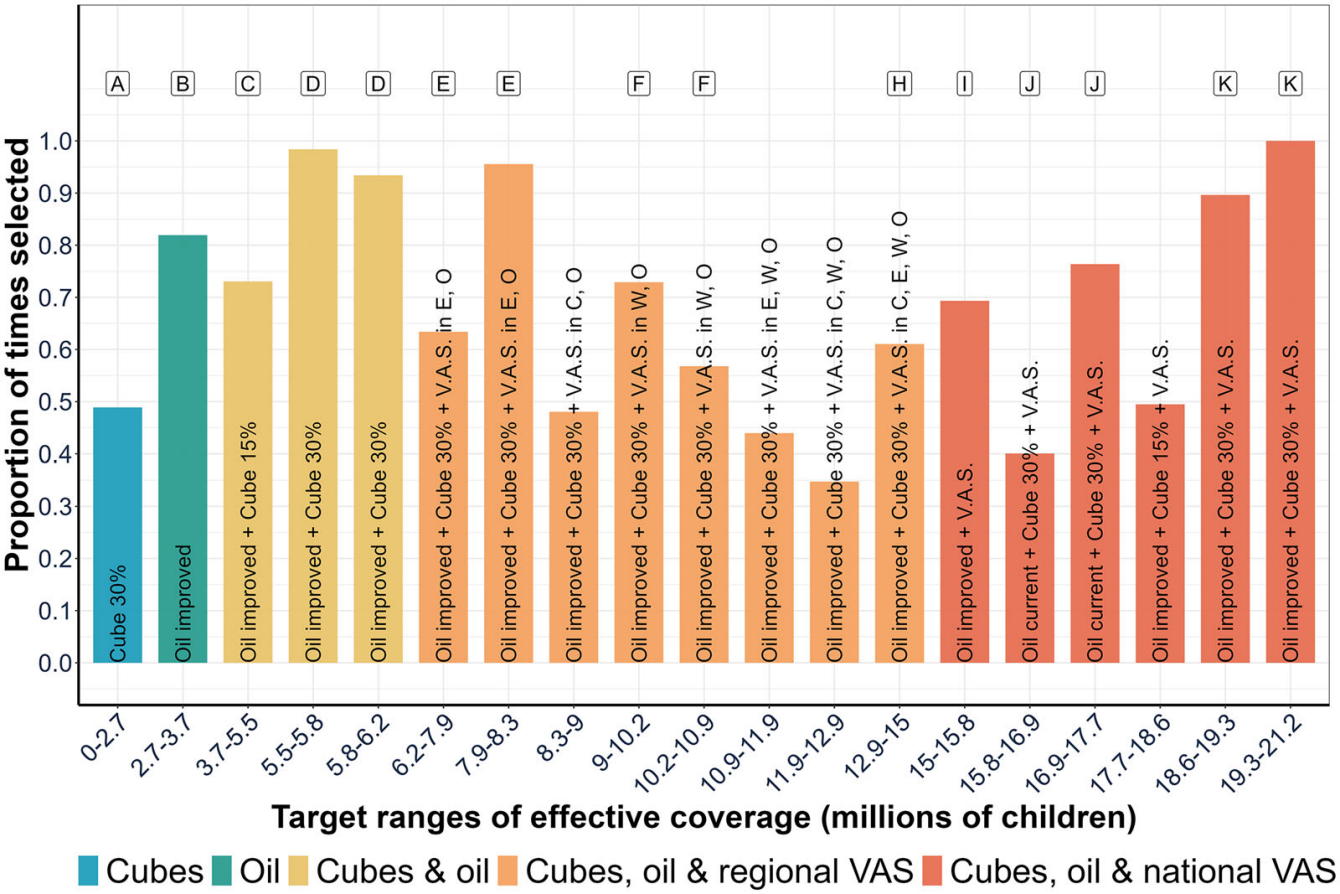
Programs and program packages most frequently deemed economically optimal, considering stochastic program costs and the number of children effectively covered. Cube 30%, fortified bouillon at 30% of Codex NRV for an adult in 2.5 g/day; oil improved, edible oils fortified with 17.5 mg/kg of VA; cube 15%, fortified bouillon at 15% of Codex NRV for an adult in 2.5 g/day; VAS, vitamin A supplementation; E, East macro‐region; O, Ouagadougou macro‐region; W, West macro‐region; N, North macro‐region; VAS without macro‐regional designations represents a national VAS program. Labels A–K represent different economically optimal programs and program packages, as identified in Table 3.

To further assess the sensitivity of the results based on our central estimates to differences in program benefits and costs, we computed the frequency with which program and program packages remained economically optimal for a given range of effective coverage, that is, for areas around target levels of effective coverage, how certain can we be that the economically optimal programs and program packages remain so when uncertainty is taken into consideration. To produce these results, we divided the entire range of feasible target levels of coverage into 20 quantile‐spaced bins^cc^ and then counted the proportion of times that each program or program package is deemed optimal across 480 simulations. We present the results of our calculations in Figure 5.

One of the main results emerging from Figure 5 is that, despite stochastic program costs and nutritional benefits, the ordering of the types of economically optimal programs and program packages remains the same as that of the optimal program packages under our central estimates presented in Table 3 (see the labels above the columns in Figure 5 for comparison). To highlight this result, we group the program packages into five color‐coded categories: fortified bouillon cubes (blue), improved oils (green), fortified cubes plus improved oils (yellow), fortified cubes plus improved oils plus subnational VAS programs (orange), and fortified cubes plus oil fortification plus a national VAS program (red). As reported earlier, but now including uncertainty in program benefits and costs, for low target levels of effectively covered children, fortified bouillon cubes and fortified oils are always part of the economically optimal program packages. As target levels of effectively covered children rise, regional VAS programs need to be included along with fortified bouillon cubes and the improved oils program. Finally, to have the largest impact on VA‐inadequacy, VAS programs must be implemented nationally. Another main result is that the program packages that are deemed economically optimal under our central estimates also are deemed optimal most of the time in their corresponding quantile‐spaced bin. For example, program package C consists of a program of fortified oil with improved compliance and a program of fortified bouillon cubes delivering 15% of Codex NRV. This program package is optimal to effectively cover up to 4.06 million children over 10 years under our central estimates (Table 3). Under stochastic benefits and costs, the programs that comprise program package C are also chosen most frequently (in approximately 70% of the simulations) to effectively cover between 3.7 and 5.5 million children.

**FIGURE 6:**
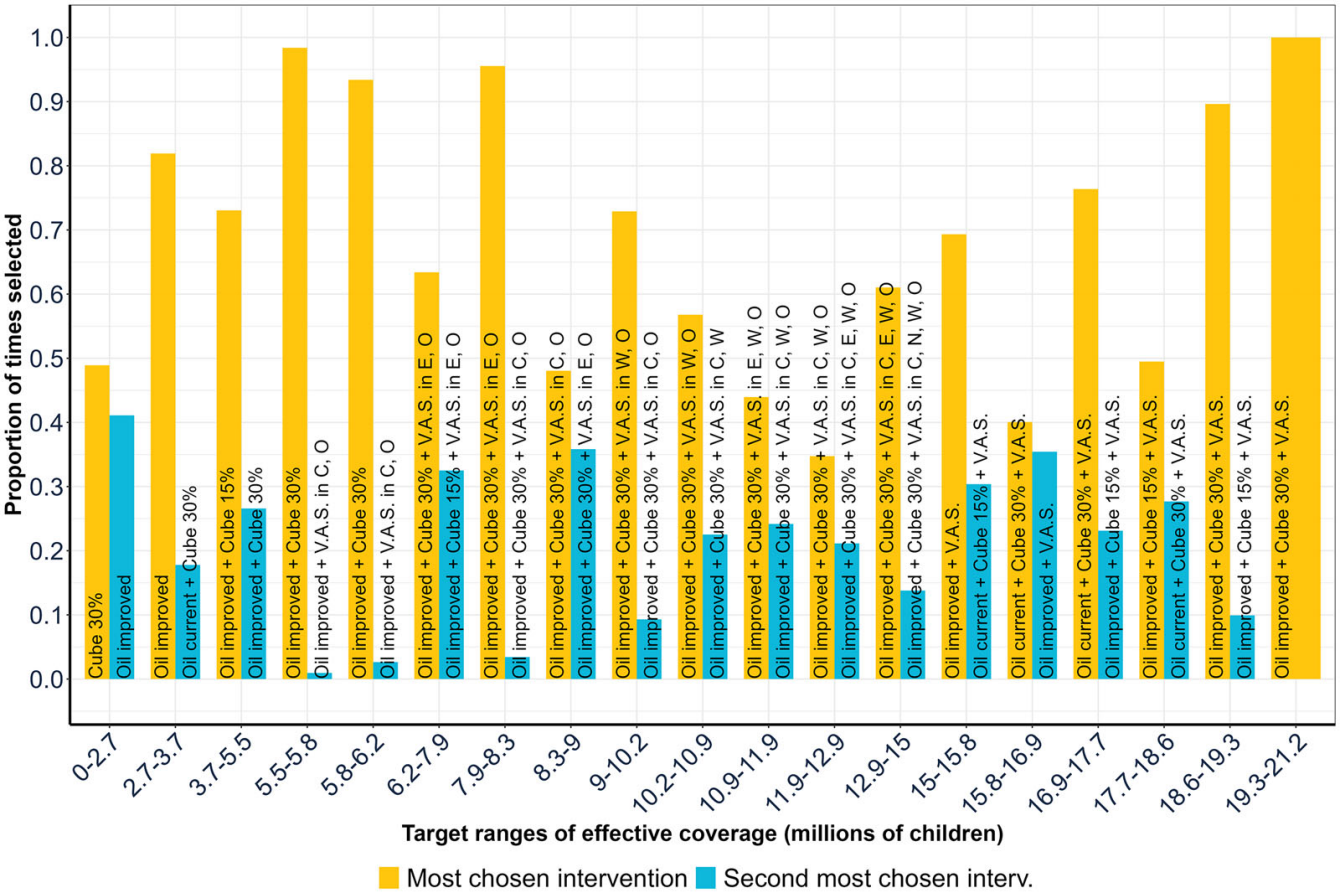
Top two programs or program packages most frequently deemed economically optimal, considering stochastic program costs and benefits. Cube 30%: fortified bouillon at 30% of Codex NRV for an adult in 2.5 g/day; oil improved: edible oils fortified with 17.5 mg/kg of VA; cube 15%, fortified bouillon at 15% of Codex NRV for an adult in 2.5 g/day; VAS: vitamin A supplementation; E: East macro‐region; O: Ouagadougou macro‐region; W: West macro‐region; N: North macro‐region; VAS without macro‐regional designations represents a national VAS program. See Table 3 for descriptions of scenario labels.

However, not all program packages deemed optimal under our central estimates (Table 3) appear in Figure 5; for example, program package G appears several times as an optimal solution but never frequently enough to make it a top choice. In addition, some of the economically optimal solutions in Table 3 appear multiple times in Figure 5, for example, program package F. To further test the robustness of programs and program packages to uncertainty in program benefits and costs, we extended the analysis to look not only at the programs and program packages that are most frequently chosen throughout the simulations, but also at those that are second most frequently chosen. We present our results in Figure 6.

The pairs of columns in Figure 6 provide further evidence that the selections of target‐specific economically optimal program packages were robust to deviations from our central estimates attributable to uncertainty in program costs and benefits. The first (yellow) column reports the proportion of times that the most frequently chosen program or program package was deemed optimal; the second (blue) column reports the proportion of times that the second most frequently chosen program package was deemed optimal; in the final column of Figure 6, all programs are deployed, so there was no second‐choice option. For example, under our central estimates (Table 3), oil improved was the optimal program package to cover between 1.1 and 2.8 million children. When we incorporate uncertainty in the benefits and costs, oil improved was the second most chosen for the range from 0 to 2.7 million effectively covered children. For the next interval, from 2.7 to 3.7 million effectively covered children, oil improved was the most chosen intervention.

The relative heights of the first and second columns provide evidence regarding how close the first and second intervention choices were to one another. In some cases, the first and second choices were fairly close, for example, for the category of 0–2.7 million children effectively covered, the proportion of times that the fortified bouillon cube program at 30% of Codex NRV was deemed optimal was only slightly higher than that for the improved edible oils program, suggesting that either of these programs would likely achieve the targeted number of effectively covered children and (importantly) do so at the lowest cost. On the other hand, in the 7.9–8.3 million range of effectively covered children, it was clear that implementing VAS in the East macro‐region would be much more likely to achieve the targeted amount of effectively covered children at the lowest cost than implementing VAS in the Center region (that choice of macro‐region was the only aspect that differentiated these two program packages). In summary, Figure 6 makes two main points. First, in the majority of the quantile‐spaced bins, the program packages that were deemed economically optimal under our central estimates (from Table 3) are also deemed economically optimal in more than 70% of the simulations, despite acknowledging uncertainty in the underlying program costs and benefits. Second, in bins in which the top choices did not have a wide margin over the second choices, the second most frequently chosen program packages comprised most of the programs in the top‐choice program package. Overall, the economically optimal vitamin A program packages for given target levels of effective coverage of vitamin A–inadequate children were robust to uncertainty regarding program‐specific benefits and costs.

### Robustness to cost perspective

Most of the costs associated with vitamin A fortification programs are paid by the citizens of LMICs, and in the context of LSFF programs, mainly by the subset of citizens who consume fortified products. Citizens contribute tax revenues to fund government investments and activities, and consumers pay higher prices for fortified foods/condiments than for their unfortified counterparts. In addition, citizens in high‐income countries contribute via tax revenues and assistance programs, generally in the form of evidence generation, policy engagement, and start‐up costs for LSFF programs. However, although citizens in LMICs make decisions at the ballot box and consumers make choices regarding food purchases, they may influence but do not make decisions regarding LSFF or VAS programs. Governments and industry (the implementing partners in all LSFF programs) make these decisions. Therefore, the results presented above focused only on the perspective that includes government and industry costs.

For completeness, we also examined the same sets of decisions using the same analytical framework from two alternative cost perspectives: (i) government‐only costs and (ii) government, industry, and premix costs. The results of these analyses are reported in online Supporting Information. Two important results arise from these analyses. First, if only government costs are considered (Table S3 and Figure S1), then the fortified bouillon cube program at 15% of Codex NRV was never chosen, but the pattern of economically optimal vitamin A programs and program packages remained basically unchanged vis‐à‐vis the core results presented above. This result is attributable to the unchanging start‐up and monitoring and evaluation costs faced by the government if the fortified bouillon cube program shifts from 15% to 30% of Codex NRV; industry costs do increase, mainly to manage the larger and more expensive premix flows, but from a government‐only cost perspective, these costs do not matter. Second, if all costs were considered, including premix costs that are a significant driver of total bouillon fortification program costs that likely will be paid by consumers in the form of higher product prices, then VAS programs entered earlier in the sequence of economically optimal program packages. In this case, bouillon fortification programs at any level of fortification were economically optimal choices only at very high levels of targeted effective coverage (Table S4 and Figure S2). The choice of cost perspective would be determined, in part, by which stakeholder groups made vitamin A program decisions (as noted above) and which groups received the benefits of vitamin A programs (consumers of fortified products). Although national decisionmakers are concerned about consumers’ disposable income and how their actions might influence it, and civil society does have a voice in micronutrient policy discussions, we chose to focus attention on government and industry and the costs that they would face because these are the stakeholder groups that ultimately choose and design vitamin A programs.

## DISCUSSION

Vitamin A deficiency remains a public health problem in many LMICs; inadequate dietary vitamin A intake is a fundamental cause. This paper presents a new tool for assisting planners in identifying cost‐effective sets of vitamin A programs and program packages to achieve alternative levels of reductions in dietary vitamin A inadequacy among children in Burkina Faso.

Our contributions build on the work of Vosti et al. and expand their work in several ways.^31^ First, the algorithm solution provides the planner with economically optimal program packages for alternative target levels of effective coverage. This provides planners with estimates of the costs of effectively covering different numbers of children and hence can be useful for establishing budgets and for overall vitamin A program sustainability. Second, we improved the methodology for assessing the robustness of model outcomes to uncertainty in both program costs and benefits. We performed 480 simulations in which program costs and benefits deviated from our central estimates and computed how often program packages deemed economically optimal under our central estimates remain so. Third, we adopt a multi‐stakeholder approach to cost analysis—government costs alone, government and industry costs, and government, industry, and premix costs. We believe that the (combined) government and industry cost perspective are the most appropriate for policy discussions because these two stakeholder groups make decisions about and implement vitamin A programs, so these results are included in the main paper. However, decision‐makers may choose alternative perspectives; the results of a government‐only cost focus are very similar to those reported above, while the inclusion of premix costs would substantially reduce the cost effectiveness of fortified bouillon programs (these results are reported in online Supporting Information Section).

Burkina Faso suffers from dietary inadequacies of an array of micronutrients and some evidence has been generated on the effectiveness and cost effectiveness of addressing some of them for the case of zinc.^5^, ^36^ This paper focuses on vitamin A. Burkina Faso currently has one vitamin A LSFF program in place, refined edible oils, which is mandated to deliver 17.5 mg/kg of vitamin A, but it performs poorly (only 39% of oils were fortified at all, and those fortified reached 85% of standard, on average).^5^ Considering dietary sources of vitamin A alone, 94% of children were estimated to be vitamin A inadequate; the existing vitamin A–fortified edible oils program brought that to 91%. Therefore, vitamin A inadequacy among young children remains a very serious public health problem. A formal network of government agencies, headed by the Ministry of Health and including the National Fortification Alliance, is in place to identify and implement programs to help reduce micronutrient inadequacies in general, with substantial support from international NGOs. A bouillon fortification Country Working Group (CWG) was formed to assess the potential for fortified bouillon to contribute to reducing inadequacies in vitamin A, iron, zinc, vitamin B12, and B9. The economic optimization results presented above, along with other modeled evidence, were delivered to the CWG as input into their deliberations.^37^

Several key messages emerge. First, as one would expect, the higher the target level of vitamin A inadequacy reduction, the higher the funding required to achieve that objective. This paper provides empirical evidence of this relationship in the context of Burkina Faso, with particular focus on specific efficient and increasingly impactful vitamin A fortification and supplementation programs. Second, LSFF programs, especially the improved edible oils program and the bouillon cube fortification program at 30% of Codex NRV in 2.5 g, formed the core set of programs for addressing vitamin A inadequacy among young children; that is, from the government plus industry cost perspective, if funds are limited, the LSFF programs are the most cost‐effective programs for addressing vitamin A inadequacy. However, if costs borne by consumers are considered, then VAS programs become more attractive than LSFF programs in funding‐constrained situations. Third, to reach higher vitamin A inadequacy reduction targets, macro‐regional VAS programs would have to be layered upon the cost‐effective base of LSFF programs. Fourth, the cost effectiveness of economically optimal program packages varied depending on targeted effective coverage levels. That is, although all interventions reported above were the least‐cost programs or program packages to achieve given levels of effective coverage, the most cost‐effective program package was comprised only of LSFF programs and effectively covers only ∼5.7 million children over a 10‐year time period, which is a small fraction of the vitamin A–inadequate population of children. Sixth, the results reported here were robust to the levels of uncertainty in program nutritional benefits and costs included in this model. Finally, but importantly, even if the most impactful programs included in this modeling exercise were deployed, vitamin A inadequacy among children in Burkina Faso would remain a serious public health problem. Therefore, improvements in the existing macro‐regional VAS programs are needed, as are new vitamin A delivery programs.

The research reported here has several limitations that may affect the results and their extrapolations to other national contexts and other micronutrient inadequacies. First, Burkina Faso was the only country included in this analysis to date. Similar modeling exercises undertaken in other geographies and on other populations may generate different patterns of results, especially if dietary intake patterns and the reaches of fortifiable food/condiment vehicles differ from those in Burkina Faso. Second, this study relied on household‐level data to estimate apparent dietary intake, levels of vitamin A inadequacies, and the effects of fortification and supplementation programs on levels of vitamin A inadequacies. Although the general comparability of results based on primary and secondary data has been demonstrated in the context of Cameroon, further work in Burkina Faso and elsewhere is required to confirm the robustness of analyses based on secondary data for the policy questions addressed here.^12^ Third, this analysis focused exclusively on vitamin A and on children 6–59 months of age. Although we expect the results for nutritional adequacy in vitamin A for other beneficiary groups, for example, women of reproductive age, to be similar to those reported for young children, results for other micronutrients and other beneficiary groups could be different. Fourth, the nutritional needs/benefits and vitamin A program cost models are designed to reflect a 10‐year planning time horizon. Many important parameters in each model are held constant over the entire time horizon. Most notably, although diets vary considerably across households, for given households, diets are assumed to remain constant over the simulation period. Key cost model parameters, for example, wage rates, are also constant over time. The only time‐variant elements of either model were the national and subnational populations' size and age distribution. All that said, robustness tests reported here suggest that uncertainty in program costs and nutritional benefits did not greatly affect model outcomes or the policy messages derived from them. Fifth, we limited modeled vitamin A bouillon cube fortification to 30% of Codex NRV in 2.5 g; higher vitamin A levels might be technically and commercially feasible, and they would further reduce vitamin A inadequacy. Sixth, due to data limitations, we were not able to model potential improvements in VAS programs; in the short term, improvements in these programs may have great scope for reducing vitamin A inadequacy among children, but these benefits would not accrue to other segments of the population. Seventh, it is possible to model the planner's problem as maximizing effective coverage for a given budget allocation rather than minimizing the cost of achieving alternative levels of effective coverage (the approach taken here). We would expect the results of this alternative approach to be very similar to those presented here, and the policy messages, which prioritize investments in LSFF programs and then the efficient overlaying on them of macro‐regional VAS programs, to be identical.^34^ Finally, we explored a fairly limited number of potential food/condiment vehicles for delivering vitamin A to young children. Future work could explore additional vehicles (e.g., fortified wheat flour or fortified rice) and would produce a larger list of policy options for addressing vitamin A inadequacy.

## CONCLUSIONS

The economic optimization tool we propose can provide planners with estimates of the nutritional benefits and the costs of efficient vitamin A programs and program packages along a continuum of objectives associated with reducing vitamin A inadequacies among children. So, rather than taking a program‐specific focus, planners could identify alternative desired target levels of vitamin A inadequacy reductions and know, for each target level, the most cost‐effective combination of current, improved, and/or new programs to implement, and what the cost of implementation would be over a 10‐year planning time horizon. Finally, the model highlighted the difference between cost‐effective program packages associated with specific levels of vitamin A inadequacy reduction and the most cost‐effective program package among all program packages, which was found to be comprised only of LSFF programs that generated a level of vitamin A inadequacy reduction that may not match planners’ objectives.

For Burkina Faso, vitamin A inadequacy among children is a serious public health problem that the current edible oils fortification program contributes little to resolve. Vitamin A–fortified bouillon at 15% of Codex NRV in 2.5 g could significantly reduce vitamin A inadequacy; increasing the level of vitamin A fortification to 30% of Codex NRV in 2.5 g would contribute even more. Indeed, the economic optimization model routinely selected bouillon fortification as a core LSFF program, along with an improved edible oils fortification program, regardless of planners’ targeted levels for reducing vitamin A inadequacies. However, due to very micronutrient‐poor diets and relatively low levels of bouillon consumption in Burkina Faso (vis‐à‐vis other West African countries), the potential contributions of fortified bouillon cubes at modeled fortification levels were muted. Finally, even if all of the most impactful modeled vitamin A programs were implemented, including a national VAS program at current coverage levels, vitamin A inadequacy among children would be reduced by only ∼50%; therefore, improvements to existing programs and additional vitamin A delivery programs are needed.

## AUTHOR CONTRIBUTIONS

A.R.C., K.P.A., A.M., R.E.‐S., and S.A.V. designed the study and developed the methods. K.P.A., K.O.‐B., and M.J. analyzed the nutritional needs/benefits and program cost data and provided the summary measures of nutritional benefits and program costs for use in the economic optimization model. A.R.C. performed all of the model simulations, with assistance from A.M.; A.R.C. and S.A.V. prepared a first draft of the paper. All authors contributed to the interpretation of simulation results and revisions of the manuscript and read and approved the final manuscript.

## CONFLICT OF INTEREST STATEMENTS

The authors declare no competing interests.

### PEER REVIEW

The peer review history for this article is available at: https://publons.com/publon/10.1111/nyas.15290

## Supporting information



Supporting Information

## Footnotes

a Because the marginal benefits of any given program depend on the contributions of other programs being considered, separate nutritional benefit model simulations must be run to estimate the benefits associated with each program and program package.^5^, ^12^

b Given Burkina Faso's 13 regions and the multiple possible program choices for each region (4 possible vitamin A LSFF programs and 1 VAS program; all are dichotomous choices), the number of combinations became computationally intractable with our current computing resources.

c Here and throughout, estimates of vitamin A inadequacy in young children are based on the nutrient density of the household diet compared to the critical vitamin A density of children, estimated using household food consumption data from the 2018‐2019 Enquête Harmonisée sur les Conditions de Vie des Ménages (EHCVM).^14^

d The LSFF programs included in this analysis would certainly deliver nutritional and perhaps other benefits to other segments of the Burkinabe population, for example, to women of reproductive age (we ignore these benefits). VAS programs directly benefit only those young children reached by them.

e This is the assumed upper bound for LSFF program performance in Burkina Faso, given porous borders, and so forth

f Throughout this paper, we use the terms bouillon cubes and cubes interchangeably.

g It is worth noting that households automatically opt in to food fortification programs via their dietary habits. The same is not true for VAS programs, which require caregivers to choose to deliver children to nurses and other professionals who administer the appropriate vitamin A capsules. Campaign‐based programs often deliver capsules to household's doorsteps, minimizing the time required by caregivers. Other VAS delivery programs sometimes require substantial amounts of caregivers’ time. Although we acknowledge these choices, we did not account for them explicitly, as they are considered to be contained in the coverage data.

h Due to start‐up periods for improved and new programs, the improved edible oils and bouillon cube fortification programs, respectively, begin to generate benefits in year 2 and year 3 of the modeling time horizon.

i It should also be noted that government and industry always jointly design LSFF programs, thereby further justifying this particular cost lens. Two other perspectives (government‐only costs and all costs, including premix costs) are addressed in online material and are summarized below.

j Caregiver costs can be substantial, especially for those in rural areas who have to travel long distances to fixed‐point VAS distribution centers, for example, clinics. However, we do not expect this omission from VAS platform cost calculations to bias model results (economically optimal program choices), as coverage rates for VAS programs already reflect caregivers’ decisions. Moreover, excluding caregiver costs is parallel to excluding premix costs, which we do in the results reported here.

k Given the 10‐year planning time horizon built into all models, we assume that on average over that 10‐year period 10% of bouillon cubes consumed in Burkina Faso would be produced domestically.

l VAS programs do not function perfectly; macro‐region‐specific coverage estimates for campaign‐based VAS distribution were used to estimate the nutritional benefits of these programs. Given the scarcity of information on the types, amounts, and impacts of investments to improve VAS program performance, no attempt was made to model VAS program improvements.

m Total national capsule costs for 2021 were estimated to be $1,928,539 (including domestic transport, storage, and handling) and represent only 6.85% of total VAS program costs.

n We use mixed integer linear programming to find the optimal solution. Our algorithm is based on the work by Vosti et al. (2023) and Santos and Toffolo (2020).^31^, ^32^

o For this characterization of the economic optimization problem, the planner focuses on government and industry costs (combined), not premix costs. Online supplementary material presents the modeling results for a planner who focuses on government costs (alone) and (in separate simulation results) on all costs (government plus industry plus premix).

p Although it is true that the clinic‐based and campaign‐based VAS programs are commonly part of the overall status quo in Burkina Faso, for this exercise, we focus on the single status quo vitamin A fortification program and then layer on additional fortification and supplementation programs.

q There are stakeholders who would prefer to maintain the status quo, for example, millers managing underperforming fortification programs and therefore reduced premix import costs and factory quality control costs. These costs are explicitly included in the scenarios assessing the costs and nutritional benefits of fortification program improvements.

r To learn more about the programs available in the menu, see **Table** **2** and Table S1.

s For example, an impactful vitamin A program will necessarily reduce the pool of VA‐inadequate children available to be addressed by other vitamin A programs.

t There may be some economies of scale in terms of adding LSFF programs and of scaling up VAS programs. We expect these cost savings to be very small relative to the main cost drivers (premix costs for LSFF programs, and staff time and logistics for campaign‐based VAS programs), so they are not considered here.

u Another way to characterize the planner's problem is to identify the program package that maximizes the number of effectively covered children for a given level of available funding. The results of such a characterization are expected to be similar to those presented here, although additional technical complexities would have to be addressed to derive these results (see, e.g., Guiges et al. 2023).^34^

v Policymakers can, of course, skip over some of these more granular policy targets, for example, jumping to 5% or 10% effective coverage. However, the more granular approach can be useful in identifying program package “tipping points” that might have funding, political, or other implications.

w Indeed, economic and other shocks can also disrupt food fortification and supplementation programs, especially in the contexts of LMICs. For example, closing international borders will impede flows of premixes and vitamin A capsules, bringing to a halt any nutritional benefits associated with such programs. This model was not designed to incorporate such extreme events, but we believe that the sensitivity analyses undertaken were broad enough in terms of nutritional benefits and program costs to incorporate expected variations in the context of Burkina Faso.

x The term *central expectations* refers to our central estimate of program‐specific and combined‐program‐specific benefits and costs over the 10‐year horizon, based on the data and assumptions that we have included in each model. These assumptions could be wrong in year 1 and/or change over 10 years. Sensitivity analyses assess the robustness of economic optimization model choices to model data and parameter assumptions.

y Formally, let $z_{c}$ be a vector of dimensions $(k^{N}+k^{s})\;\times 1$ whose elements are independent realizations of N(0,0.1); this vector represents changes from our central cost estimates that could occur on an annual timestep due to unforeseen developments in the future, for example: a change in the exchange rate could make some of the costs of imported inputs more expensive beginning in year 5 of the planning horizon, thereby increasing the total 10‐year costs of all programs making intensive use of imported inputs. In each round of simulations, we multiplied our annual central estimates of costs by $1+z_{c}$ at the program level and then aggregate the costs at the program package level over the planning time horizon (10 years). We did something similar for the program benefits and multiply them by $1+z_{b}$, where $z_{b}$ is a different $(k^{N}+k^{s})\;\times 1$ vector of independent realizations of N(0,0.1)⟨/END⟩

z Technically, the unit of measure for benefits is child‐years of effective coverage, as we do not know precisely which children in the population will transition from vitamin A inadequacy to adequacy in any given year; indeed, many of the same children might be “re‐achieving” adequacy each year, but this is not an issue of double‐counting, as removing programs at any point in time would cause some children to transition back to vitamin A inadequacy.

aa Note that the current oil program (oil curr) can serve as a benchmark against which other programs can be measured, in Figure 2 in terms of nutritional benefits generated and in terms of program costs.

bb Technically, this is the maximum number of children effectively covered by each program or program package, as this represents the upper bound of the interval of children effectively covered by each program or program package, after which the economic optimization algorithm would tip to the next vitamin A program or program package. A total of 40 such intervals were used in the analytical process. For example, if a planner chose 1.0 million children effectively covered as the policy objective, scenario A (Cube 30%) would be chosen, but that choice would effectively cover 0.1 million *more* children than the policy objective. Note that the changes between one row and the next row below of column (4) are not of equal breadth as the nutritional benefits associated with adding some programs will be larger than for others.

cc Quantiles are cut‐points that divide the range of any distribution into continuous intervals with equal probabilities. In our context, the simulations yield a distribution of tipping points, that is, levels of effective coverage that require different programs or program packages to achieve. We divided the range of this distribution into 20 quantiles, such that the space between each quantile, or bin, had roughly the same number of programs or program packages. To compute the proportion of times a program or program package is deemed economically optimal within a range, we chose quantile‐spaced bins as the criterion for determining the length of ranges over which the proportions would be computed. We chose quantile‐spaced bins, as opposed to the more traditional evenly‐spaced bins, to account for the clustering of outcomes around tipping points. That is, the set of all optimal choices was not evenly spaced throughout the range of effective coverage levels. If we divided the whole range into 20 evenly‐spaced bins, we would have overrepresented regions with no tipping points, and underrepresented regions of the distribution where the tipping points were occurring. See Cattaneo et al. (2019) for details regarding quantile‐spaced bins.^35^
