# Supplementary material for: Cost effectiveness of fortified bouillon in addressing Burkinabe children's vitamin A inadequacy: An economic optimization model
Source: Ann N Y Acad Sci. 2025 Feb 5;1544(1):143–58. doi: 10.1111/nyas.15290 (PMC11829321; doi:10.1111/nyas.15290)
Supplement: Supplementary file 1 — Supporting Information [file NYAS-1544-143-s001.docx]

**Cost-effectiveness of fortified bouillon in addressing Burkinabe children’s vitamin A inadequacy:**

**An economic optimization model**

**On-line Supplementary Material**

This document contains the supplementary material to support the main paper reporting the results of research to identify the economically optimal combinations of vitamin A intervention programs in Burkina Faso.

***Details of the Modeled Food/Condiment Fortification Programs***

**Table SM.1** reports the details of the modeling parameters associated with the vitamin A content of fortified foods. More specifically, the second column reports the amounts of vitamin A either mandated by standard for edible oils or assumed to be included in fortified bouillon cubes (no bouillon fortification standards currently exist in Burkina Faso). The final two columns of **Table SM.1** report assumptions regarding the proportion of fortifiable foods that are fortified at *any* level, and the level of fortification (vis-à-vis the standard for oil or the assumption for bouillon cubes) for those foods that *are* fortified.

| **Table SM.1**: Large-scale vitamin A food/condiment fortification program labels and modeling parameters | | | |
| --- | --- | --- | --- |
|  |  | ***Modeled Compliance with Standard*** | |
| **Food/Condiment Vehicle Label** | **National Fortification Standard*** | **Fortifiable Food/Condiment Vehicles Fortified to any Extent***** | **Average Fortification Level among Fortified Food/Condiment** |
|  | *(mg/kg)* |  | *(Pct. of national standard)* |
| Oil current | 17.5 | 39% | 85% |
| Oil improved | 17.5 | 75% | 100% |
| Cube 15%^**^ | 48 | 75% | 100% |
| Cube 30%^**^ | 96 | 75% | 100% |
| ^*^ The standard for oil is based on Burkina Faso’s existing refined oil fortification standard; the national standards for bouillon cubes are hypothetical. | | | |
| ^**^ Micronutrient addition levels (at point of consumption) to meet 15% and 30% of Codex Nutrient Reference Value for VA, respectively, in 2.5 g bouillon per day, corresponding to 48 mg vitamin A per kg of bouillon.  *** Global Alliance for Improved Nutrition, 2018^15^ | | | |

***Accounting for the Costs and Nutritional Benefits of Alternative Vitamin A Supplementation Programs***

One challenge we faced was integrating the (incomplete) vitamin A supplementation (VAS) program coverage data from multiple sources into the nutritional benefits and the cost models. **Table SM.2** reports VAS coverage estimates, for the two VAS delivery platforms currently in place in Burkina Faso; bi-annual national child health campaigns and routine VAS at health clinics. VAS coverage via clinics is primarily relevant for 9-month-old children who are scheduled to received measles vaccines at that point and who generally also receive one (100,000 IU) dose of VAS. To estimate the number of capsules required (and their cost) for clinic-based VAS distribution, we estimated that 20% of children 6-11 months of age were 9 months of age. Technically, this would lead to a very small double-counting of capsule costs, since a fraction of children receiving VAS from clinics would also be ‘covered’ by the national VAS campaigns, but these double-counted children would only receive one capsule per year, compared to the two capsules per year that the older group would receive. To get a sense for the cost accounting consequences of this double-counting, we estimate that ~$1,500 per year would be spent on capsules for 9-month-old children via VAS distributed at clinics.  To estimate the nutritional benefits of VAS, only the coverage data on national campaigns were used. Therefore, our cost-effectiveness estimates likely include a small amount of double counting for capsule costs, and a small undercounting of the nutritional benefits for children 9 months of age (the coverage estimates used in our calculations were slightly lower than the measles vaccination coverage rates). Neither of these differences were deemed large enough to make any difference in the results of the optimization simulations, and fall well within the range of uncertainty tested in the context of sensitivity analyses.

**Table SM.2**. High-dose vitamin A supplementation coverage estimates among children 6-59 months of age

| **Region** | **VAS coverage via national campaign (%)** | | | **VAS coverage via clinic visit (%)** | |
| --- | --- | --- | --- | --- | --- |
|  | **Urban** | **Rural** | **Source** |  | **Source** |
| Boucle du Mouhoun | 44% | 77% | [1] | 90% | [3] |
| Cascades | 44% | 77% | [1] | 94% | [3] |
| Centre | 44% | 77% | [1] | 86% | [3] |
| Centre-Est | 44% | 77% | [1] | 89% | [3] |
| Centre-Nord | 44% | 77% | [1] | 83% | [3] |
| Centre-Ouest | 71% | 89% | [2] | 88% | [3] |
| Centre-Sud | 72% | 91% | [2] | 93% | [3] |
| Est | 44% | 77% | [1] | 72% | [3] |
| Hauts Bassins | 83% | 94% | [2] | 65% | [3] |
| Nord | 44% | 77% | [1] | 76% | [3] |
| Plateau-Central | 94% | 88% | [2] | 86% | [3] |
| Sahel | 44% | 77% | [1] | 69% | [3] |
| Sud-Ouest | 86% | 91% | [2] | 73% | [3] |

Coverage sources:

[1] Report of the evaluation survey of the national post-campaign coverage of Vitamin A Supplementation (VAS), deworming and malnutrition screening, 2018.

[2] Post coverage assessment survey of National Vitamin A Supplementation Days, second round, 2020.

[3] Measles vaccination coverage rates from Enquête Démographique et de Santé du Burkina Faso 2021.

***Results of Economic Optimization Considering Government Costs Only***

The main paper reports the results that emerge from the economic optimization algorithm when both government and industry costs associated with LSFF programs were considered; premix costs were omitted but the industry costs associated with managing the purchase, transportation, storage, etc. of premixes were included at 5% of the value of premix. In what follows, we report the results obtained in two additional model simulations. The first set of results considered *only* the government costs associated with each LSFF program; the second set of results considered *all* costs for all stakeholders, including the premix costs.

**Table SM.3** reports the results that emerge from the economic optimization algorithm if only government costs are considered. Comparing these results with those that appear in **Table 3** of the main paper, several points merit mention, although it is important to recall that the economically optimal sets of programs are somewhat different in each case. First, the government costs (alone) of managing LSFF programs is much lower than that of industry. For example, the (baseline) current oil program costs government approximately $0.8m over 10 years while costing industry approximately $1.4m ($2.2m-$0.8m) over the same period. Second, since government costs associated with monitoring and evaluation of LSFF programs do not vary with the amounts of fortificants included in premixes (the same number and types of tests, etc., are performed), when viewed from the perspective of government costs alone, the economic optimization algorithm never chose Cube 15%. Third, governments were assumed to cover the full costs of all VAS programs, so once VAS programs are efficiently introduced, the differences between the **Table 3** and **Table SM.3** results regarding impacts, costs, and cost-effectiveness were greatly reduced.

| **Table SM.3**: Economically Optimal vitamin A intervention programs for different target levels of effective coverage; *Government costs only*, over 10 years |
| --- |
| \| **Simulation Labels** \| **Optimal vitamin A Intervention Programs** \| **VAS Regions^†^** \| **Children Effectively Covered** \| \| **Total cost^*^** \| **Cost/child Effectively Covered** \| \| --- \| --- \| --- \| --- \| --- \| --- \| --- \| \| *(1)* \| *(2)* \| *(3)* \| *(4)* \| *(5)* \| *(6)* \| *(7)* \| \|  \|  \|  \| *Pct. of pop.^‡^* \| *Millions* \| *millions of USD* \| *USD* \| \| A0 \| Oil current \|  \| 2.0% \| 0.7 \| 0.8 \| 1.2 \| \| A \| Oil current \|  \| 2.0% \| 0.7 \| 0.8 \| 1.2 \| \| B \| Oil improved \|  \| 7.8% \| 2.8 \| 1.6 \| 0.6 \| \| C \| Cube 30% + Oil improved \|  \| 15.9% \| 5.7 \| 2.6 \| 0.5 \| \| D \| Cube 30% + Oil improved + VAS \| E, O \| 24.3% \| 8.7 \| 10 \| 1.1 \| \| E \| Cube 30% + Oil improved + VAS \| C, E \| 30.1% \| 10.8 \| 12.8 \| 1.2 \| \| F \| Cube 30% + Oil improved + VAS \| C, N \| 33.5% \| 12.0 \| 14.6 \| 1.2 \| \| G \| Cube 30% + Oil improved + VAS \| C, E, N, O \| 41.9% \| 15.0 \| 22.1 \| 1.5 \| \| H \| Cube 30% + Oil improved + VAS \| C, E, W, O \| 44.4% \| 15.9 \| 24.4 \| 1.5 \| \| I \| Cube 30% + Oil improved + VAS \| C, N, W, O \| 47.7% \| 17.1 \| 26.2 \| 1.5 \| \| J \| Cube 30% + Oil improved + VAS \| C, E, W, O, N \| 53.6% \| 19.2 \| 30.7 \| 1.6 \| \| ^†^ The labels C, E, W, O, N in column 3 represent the macro-regions of Center, East, West, Ouagadougou, and North, respectively. The absence of a macro-regional label in column 3 indicates that no VAS program was deemed optimal; the presence of *all* macro-regional labels indicates a national vitamin A supplementation (VAS) program was deemed optimal. \| \| \| \| \| \| \| \| ^‡^ Calculated with respect to the population of VA-inadequate children. \| \| \| \| \| \| \| \| ^*^ All costs are reported in 2021 USD. \| \| \| \| \| \| \| |

The patterns of total program costs and cost-effectiveness of economically optimal sets of programs are similar across the government-cost-only (**Figure SM.1**) and government-plus-industry perspectives (**Figure 3** in the main paper). As indicated above, from the government-cost-only perspective, LSFF program costs are relatively low (first several points of **Figure SM.1a** and **Figure 3a**), and then increase markedly as VAS programs are layered onto LSFF programs (remaining sets of points in Figure SM.1a and Figure 3a). Cost-effectiveness patters are also roughly similar (comparing **Figure SM.1b** and **Figure 3.b**), but the levels of cost per effectively covered child tend to be somewhat lower from the government-cost-only perspective at each level of effective coverage.

**Figure SM.1:** Costs and cost-effectiveness of economically vitamin A intervention programs; *government costs only,* over 10 years

1.
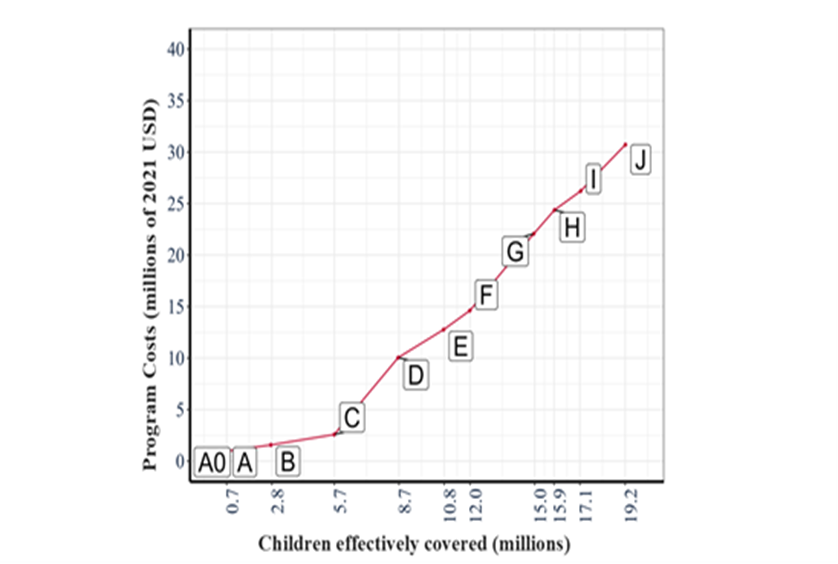
Total costs of economically optimal programs and program packages

Note: Effective coverage refers to the number of children who achieve dietary adequacy in vitamin A due to simulated programs or program packages.

1.
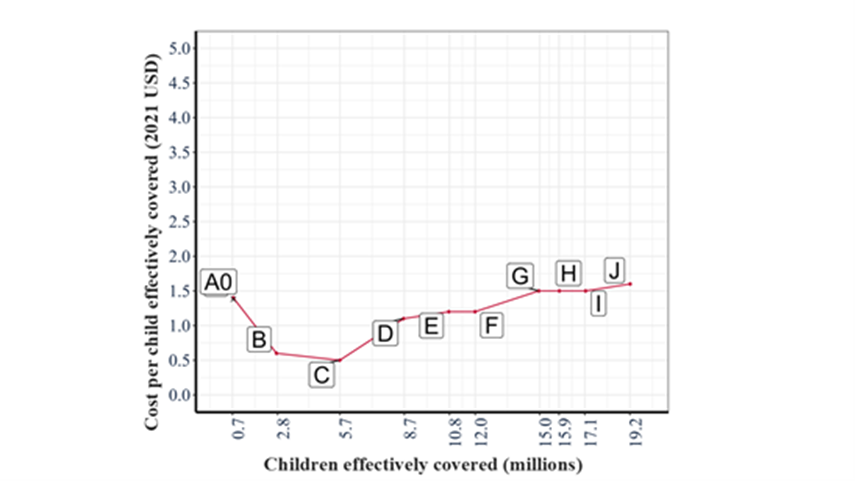
Cost-effectiveness of economically optimal programs and program packages

Note: Effective coverage refers to the number of children who achieve dietary adequacy in vitamin A due to simulated programs or program packages.

***Results of Economic Optimization Considering Government, Industry, and Premix Costs***

Recall that in the other two optimization perspectives (government-only and government-plus-industry) premix costs are assumed to be paid by consumers and hence are not considered. **Table SM.4** reports the results that emerge from the economic optimization algorithm if *all* vitamin A intervention program costs (government costs plus industry costs plus premix costs) are considered. Comparing these results with those that appear in **Table 3** of the main paper, several points merit mention. First, if decision-makers consider premix costs, then the hypothetical bouillon fortification programs delivering 15% and 30% of Codex NRV of VA, respectively, were the most costly and hence the last two programs to be implemented. Second, if premix costs are considered, the cost per child effectively covered increased dramatically, for combinations of optimal programs along the children-effectively-covered continuum.

| **Table SM.4**: Economically Optimal vitamin A intervention programs for different target levels of effective; Government, industry, *and* premix costs, over 10 years |
| --- |
| \| **Simulation Labels** \| **Optimal vitamin A Intervention Programs** \| **VAS Regions^†^** \| **Children Effectively Covered** \| \| **Total cost^*^** \| **Cost/Child Effectively Covered** \| \| --- \| --- \| --- \| --- \| --- \| --- \| --- \| \| *(1)* \| *(2)* \| *(3)* \| *(4)* \| *(5)* \| *(6)* \| *(7)* \| \|  \|  \|  \| *Pct. of pop.^‡^* \| *Millions* \| *Millions of USD* \| *USD* \| \| A0 \| Oil current \|  \| 2.0% \| 0.7 \| 3.5 \| 4.7 \| \| A \| VAS \| O \| 3.3% \| 1.2 \| 3.0 \| 2.5 \| \| B \| Oil improved \|  \| 7.8% \| 2.8 \| 7.8 \| 2.8 \| \| C \| Oil current + VAS \| C, E \| 10.3% \| 3.7 \| 14.2 \| 3.8 \| \| D \| Oil improved + VAS \| C, O \| 15.9% \| 5.7 \| 16.5 \| 2.9 \| \| E \| Oil improved + VAS \| W, O \| 19.8% \| 7.1 \| 19.4 \| 2.7 \| \| F \| Oil improved + VAS \| C, W, O \| 24.8% \| 8.9 \| 25.1 \| 2.8 \| \| G \| Oil current + VAS \| C, E, W, O, N \| 35.7% \| 12.8 \| 32.2 \| 2.5 \| \| H \| Oil improved + VAS \| C, E, W, O, N \| 43.2% \| 15.5 \| 35.9 \| 2.3 \| \| I \| Oil improved + Cube 15% + VAS \| C, E, W, O, N \| 49.9% \| 17.9 \| 47.0 \| 2.6 \| \| J \| Oil improved + Cube 30% + VAS \| C, E, W, O, N \| 53.6% \| 19.2 \| 56.7 \| 3.0 \| \| ^†^ The labels C,E,W,O,N in column 3 represent the macro-regions of Center, East, West, Ouagadougou, and North, respectively. The absence of a macro-regional label in column 3 indicates that no VAS program was deemed optimal; the presence of *all* macro-regional labels indicates a national VAS program was deemed optimal. \| \| \| \| \| \| \| \| ^‡^ Calculated with respect to the population of VA-inadequate children. \| \| \| \| \| \| \| \| * All costs are reported in 2021 USD. \| \| \| \| \| \| \| |

Finally, **Figure SM.2** depicts total costs and cost per effectively covered child for the baseline (current oil) and all of the economically optimal sets A through J of vitamin A intervention programs. Once again, comparisons with the companion **Figure 3** in the main paper, bring out a few key points. First, program costs were higher and more linearly related to the number of children effectively covered – this is to be expected since premix costs represent more than 90% of all LSFF programs and the addition of the premix-cost-heavy hypothetical bouillon fortification programs in the final two scenarios dramatically increases costs. Second, as noted above, the absolute cost per child effectively covered increased substantially from this full-cost perspective. Finally, the pattern of cost-effectiveness in **Figure SM.2** is much different from that of **Figure 3** of the main paper, primarily because VAS programs were introduced earlier in the optimization set sequence and in somewhat different spatial patterns.

**Figure SM.2:** Solution to the optimization problem considering start-up, operational, and premix costs

1.
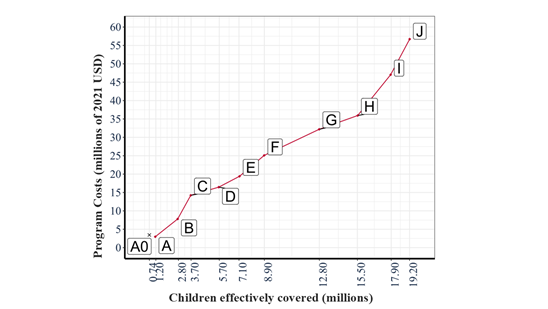
Total costs of economically optimal programs and program package

Note: Effective coverage refers to the number of children who achieve dietary adequacy in vitamin A due to simulated programs or program packages.

1.
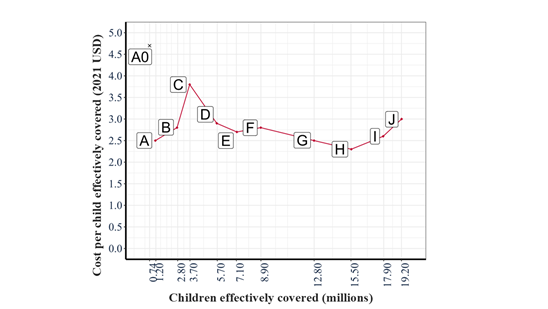
Cost-effectiveness of economically optimal programs and program packages

Note: Effective coverage refers to the number of children who achieve dietary adequacy in vitamin A due to simulated programs or program packages.
